# Nutritional Care for Adults With Obesity Treated With Glucagon-Like Peptide (GLP)-1 and Dual Glucose-Dependent Insulinotropic Polypeptide (GIP)/GLP-1 Receptor Agonists: A Review of Evidence, Gaps, and Clinical Implications

**DOI:** 10.7759/cureus.109731

**Published:** 2026-05-27

**Authors:** Cici McGuane, Nosha Farhadhar, Khavir A Sharieff, Stephanie N Petrosky, Karima Alabasi

**Affiliations:** 1 Nutrition, Nova Southeastern University Dr. Kiran C. Patel College of Osteopathic Medicine, Miami, USA; 2 Hematology and Medical Oncology, Methodist Hospital, San Antonio, USA; 3 Surgery, Nova Southeastern University Dr. Kiran C. Patel College of Osteopathic Medicine Tampa Bay Campus, Tampa, USA; 4 Nutrition, Nova Southeastern University Dr. Kiran C. Patel College of Osteopathic Medicine, Fort Lauderdale, USA; 5 Nutrition, Nova Southeastern University Dr. Kiran C. Patel College of Osteopathic Medicine, Clearwater, USA

**Keywords:** dietitian nutritionists, gip and glp-1 receptor agonist, glp-1 receptor agonists, incretin-based therapies, nutrient deficiencies, nutrition care, s: nutrition, weight loss and obesity, weight loss pharmacotherapy

## Abstract

Glucagon-like peptide‑1 receptor agonists (GLP‑1 RAs) and dual glucose‑dependent insulinotropic polypeptide/glucagon-like peptide‑1 receptor agonists (GIP/GLP‑1 RAs) have rapidly advanced obesity treatment, producing weight‑loss outcomes that exceed previous pharmacologic options. Despite their growing use, nutrition-focused clinical guidance has not kept pace, leaving practitioners responsible for supporting safe therapy implementation with gaps. This review summarizes current evidence on the nutritional implications of GLP‑1-based pharmacotherapy in adults with obesity, including common gastrointestinal adverse events, reduced energy intake, potential macro‑ and micronutrient inadequacies, and risks of lean mass loss. To address these gaps, we introduce an evidence-informed nutrition framework to guide health care professionals in assessment, diagnosis, intervention, and monitoring during GLP‑1 therapy.

A structured literature search of peer‑reviewed studies published between 2015 and 2025 was conducted using PubMed, Embase, and Google Scholar. Across randomized controlled trials, GLP‑1 and dual GIP/GLP-1 therapies produced mean weight reductions of approximately 5% to more than 20%, with gastrointestinal symptoms frequently reported and often impacting dietary tolerance. Evidence also suggests early reductions in caloric intake and inadequate consumption of several essential nutrients, while long‑term data on micronutrient status and body composition remain limited.

Findings emphasize the need for proactive, individualized medical nutrition therapy to optimize treatment tolerance, preserve lean mass, prevent nutrient deficiencies, and support sustainable outcomes. The proposed nutrition framework offers a structured clinical approach to address these challenges. Future research should focus on longitudinal dietary assessment, biomarker monitoring, and validation of standardized nutrition protocols for patients receiving GLP‑1-based obesity pharmacotherapy.

## Introduction and background

Adult obesity in the United States presents a significant public health challenge, with an estimated 41.9% of adults aged 20 years and older meeting criteria for obesity (body mass index ≥ 30 kg/m²) during the period 2017-March 2020 [1]. Notably, the prevalence of severe obesity (BMI ≥ 40 kg/m²) was approximately 9.2% in that same timeframe [1]. The condition is associated with elevated incidence of hypertension, type 2 diabetes, cardiovascular disease, and some cancers, and is both common and costly, incurring nearly US $173 billion in excess annual medical expenditures in recent estimates [1]. Given the scale of its prevalence, the magnitude of associated comorbidity burden, and the substantial economic implications, a comprehensive plan for the prevention, management, and treatment of obesity is a priority.

Over the last few years, a new class of anti-obesity medications (AOMs) has emerged, substantially transforming obesity care. Although they were developed for the treatment of type 2 diabetes (T2DM), glucagon-like peptide receptor agonists (GLP-1 RAs) and glucose-dependent insulinotropic polypeptide/glucagon-like peptide receptor agonists (GIP/GLP-1 RAs) have rapidly gained popularity and use for the treatment of obesity. Between 2019 and 2023 alone, the use of the drugs has increased by over 700% [2] in patients with overweight and obesity, with an estimated 15 million adults in the U.S. currently using a GLP-1 drug for weight loss, and an even greater number using the drug for T2DM [3]. As uptake continues to expand, due to broader access and declining costs, utilization is expected to grow substantially, particularly when considering the additional volume attributable to off‑label use. Across various pharmaceutical company-funded trials, GLP-1 RAs and GIP/GLP-1 RAs have been shown to reduce weight by 5-20%, but the results are not without side effects and complications, many of which are not yet fully understood [4]. Given the pivotal role that nutrition practitioners play in the treatment and care of adults with obesity, it is imperative that they are informed on GLP-1 RAs and GIP/GLP-1 RAs, their mechanism of action, and the evidence-based practices and protocols available to support the optimal outcomes of their clients. Understanding the mechanisms of action, systemic effects, and the spectrum of adverse events associated with GLP-1 and GIP/GLP-1 receptor agonists is essential to designing evidence-based protocols that effectively mitigate adverse events and address the potential nutrition-related concerns.

The role GLP-1 RAs and GIP/GLP-1 RAs play in reducing appetite, delaying gastric emptying, and modulating central nervous system pathways lead to reduced caloric intake, increasing the risks associated with nutrient deficiencies, loss of lean body mass, and gastrointestinal issues [4]. Given the relative novelty and rapidly increasing utilization of GLP-1 and dual GIP/GLP-1 receptor agonists in the management of adults with obesity, this narrative review aims to synthesize current evidence and identify research gaps concerning their effects on nutritional status.

History of GLP-1 RAs and dual GIP/GLP-1 RAs

Although GLP-1 RAs and dual GIP/GLP-1 RAs may seem relatively novel, knowledge of incretin gut hormones that lower postprandial glucose can be traced back to the early 1900s. In 1973, Dupré et al. identified the first incretin hormone, GIP, gastric inhibitory polypeptide, later called glucose-dependent insulinotropic polypeptide [5]. They successfully isolated the polypeptide and demonstrated its role in stimulating insulin secretion in a glucose-dependent manner [5]. In the 1980s, Mojsov, working with Habener, identified GLP-1 (glucagon-like peptide-1) as an incretin hormone [6]. Both incretin hormones are endogenously secreted in the gastrointestinal tract and exert similar effects. GIPs are secreted from the K-cells in the duodenum and jejunum, whereas GLP-1s are secreted by the L-cells near the intersection of the distal ileum and colon. As a result of the reduction or loss of the incretin effect experienced by individuals with type 2 diabetes (T2DM), [7] the therapeutic potential role of GLP-1 RA analog drugs was investigated in the late 1980s.

Nearly 20 years after their discovery, GLP-1 RAs entered the market as a pharmaceutical drug with the debut of exenatide (Byetta®) on April 28, 2005 [8]. Byetta® was indicated as an “adjunct to diet and exercise to improve glycemic control in adults with type 2 diabetes mellitus” [8]. Byetta® was a short-acting (2.4-hour half-life) GLP-1 RA derived from the venom of the Gila monster [9] that eventually led to the development of a longer-acting version linked to a fatty acid, the first of which was liraglutide (Victoza®) in 2010 [10].

In clinical trials, exenatide (approved in 2005 for T2DM) and liraglutide (approved in 2010 for T2DM) consistently demonstrated a weight loss effect, even though it was not the primary endpoint of the studies [11]. During the phase three studies of exenatide, participants reportedly lost −1.6 ± 0.3 kg over 30 weeks [11], and liraglutide also showed comparable reductions in a similar time frame [12]. These findings suggested that the weight reduction may have been independent of improved glycemia and instead mediated by the appetite suppression and delayed gastric emptying caused by the drugs.

Given these observations, Novo Nordisk pursued exploring GLP-1 RAs as AOMs. The Food and Drug Administration (FDA) has exclusive guideline thresholds regarding efficacy for any pharmaceutical drug that is seeking marketing authorization as a weight-loss agent in the United States, and a key stipulation is that weight loss must be the primary endpoint of the study trial [13]. The FDA efficacy benchmarks state that a drug is considered effective for weight reduction and maintenance in patients with obesity or overweight with comorbidities if, after one year of treatment at the maintenance dosage, the difference in mean weight reduction between the drug and control groups is at least 5% and is statistically significant [13].

With that in mind, the Satiety and Clinical Adiposity-Liraglutide Evidence (SCALE) Obesity and Prediabetes trial was launched to specifically evaluate weight reduction in adults with obesity without diabetes treated with once-daily liraglutide 3.0 mg [14]. During the 56-week trial, 63.2% of the patients treated with liraglutide lost at least 5% of their body weight, and 33.1% lost more than 10% of their body weight, an overall mean reduction of 8.4 kg (−8.0%) for the treatment group [14]. Based on these results, liraglutide (Saxenda®) was approved by the U.S. FDA on December 24, 2014, making it the first once-daily glucagon-like peptide-1 receptor agonist (GLP-1 RA) indicated as an adjunct to a reduced-calorie diet and increased physical activity for chronic weight management in adults with obesity (BMI ≥30 kg/m2) or who are overweight (BMI ≥27 kg/m2) and in the presence of at least one weight-related comorbid condition [15].

The next major development in the realm of GLP-1 RA use for weight management arose from the results of the Semaglutide Treatment Effect in People with Obesity (STEP) program published in 2021; the trial evaluated a semaglutide 2.4 mg once weekly treatment and demonstrated an average 14.9% mean weight loss over 68 weeks compared to 2.4% for the placebo group [16]. Having exceeded all previous pharmacologic weight-loss drugs, semaglutide (Wegovy®) was approved by the FDA in June 2021 [17]. Semaglutide had previously been approved for T2DM under the name Ozempic® in December 2017.

The next generation of incretin-based therapy came with the launch of tirzepatide by Eli Lilly, a dual agonist of the glucose-dependent insulinotropic polypeptide (GIP) and GLP-1 receptor agonists, approved by the FDA (under brand name Mounjaro®) on May 13, 2022, as a once-weekly injectable treatment for adults with T2DM [18]. Within a year, and after the success of the SURMOUNT clinical program targeting individuals with obesity or overweight without diabetes, [19] tirzepatide was officially approved by the FDA in November 2023 for chronic weight management under the brand name Zepbound® [20]. The results of the SURMOUNT clinical trials revealed that the tirzepatide treatment group had a reduction in body weight of 20% or more, [19], a reduction far greater than any previously observed by a pharmacologic weight loss treatment. Before GLP-1 RAs and GIP/GLP-1 RAs, the greatest weight reductions through pharmacologic treatments were observed with phentermine; the reductions were roughly half that of tirzepatide, with many more side effects [21]. As of November 2025, liraglutide (Saxenda®), semaglutide (Wegovy®), and tirzepatide (Zepbound®) are the three GLP-1 or GIP/GLP-1 therapies approved by the FDA for the indication of chronic weight management (obesity/overweight + comorbidity) in the United States, with more in development. Newer versions and variations of these drugs are focused on shifting from injectable administration to pill form, which could potentially broaden access and acceptability to a greater population. Table 1 below summarizes the key trials leading to the approvals of all three major GLP-1 and GIP/GLP-1 drugs. 

**Table 1:** GLP-1 and dual GIP/GLP-1 Approval Trial Data FDA: Food and Drug Administration, SCALE: Satiety and Clinical Adiposity—Liraglutide Evidence, STEP: Semaglutide Treatment Effect in People with Obesity, SC: subcutaneous, GLP: Glucagon-like peptide, GIP: glucose-dependent insulinotropic polypeptide.

Drug	Brand (US)	FDA approval for obesity	Approved obesity dose (adult)	Key pivotal obesity trial & population	Mean weight loss in the trial
Liraglutide	Saxenda	2014 – chronic weight management in adults with obesity or overweight + comorbidity [15]	3.0 mg SC once daily (after titration from 0.6 mg)	SCALE Obesity & Prediabetes – adults with obesity/overweight without diabetes, 56 weeks	~8% mean body-weight loss vs 2.6% with placebo (≈ 5–6 percentage-point placebo-subtracted)[14]
Semaglutide	Wegovy	2021 – chronic weight management in adults with obesity or overweight + comorbidity [17]	2.4 mg SC once weekly (titrated up over 16 weeks)	STEP 1 – adults with obesity/overweight without diabetes, 68 weeks	~14.9% mean body-weight loss vs 2.4% with placebo (≈ 12.4 percentage-point placebo-subtracted [16]
Tirzepatide (GIP/GLP-1 RA)	Zepbound	2023 – chronic weight management in adults with obesity or overweight + comorbidity [18]	5, 10, or 15 mg SC once weekly (titrated from 2.5 mg; maintenance 5–15 mg)	SURMOUNT-1 – adults with obesity/overweight without diabetes, 72 weeks	~15.0%, 19.5%, and 20.9% loss at 5, 10, and 15 mg vs 3.1% with placebo (up to ≈18 percentage-point placebo-subtracted at 15 mg)[19]

## Review

Literature review

For this narrative review, a comprehensive literature search was conducted using PubMed, Embase, and Google Scholar to identify relevant peer-reviewed articles on the use of glucagon-like peptide-1 receptor agonists (GLP-1 RAs) and nutrition-related considerations in the management, interventions, and care of adults with obesity. Key terms and Boolean operators were used in a variety of combinations throughout the search: “GLP-1 receptor agonist” OR “dual GIP/GLP-1” OR “ “glucagon-like peptide-1” OR “glucose-dependent insulinotropic polypeptide/glucagon-like peptide receptor agonists” OR “incretin” OR “semaglutide” OR “liraglutide” OR “tirzepatide” AND “obesity” OR “weight loss” OR “overweight” AND “nutrition” OR “diet” OR “dietary intake” OR “nutrient deficiencies” OR “dietitian” OR “nutrition counseling” OR “adverse events." In addition, the reference lists of the relevant articles were also screened to identify additional articles to include. Inclusion criteria consisted of peer-reviewed human studies, clinical trials, observational studies, review articles, consensus statements, and clinical guidelines that evaluated GLP-1 receptor agonists or dual GIP/GLP-1 receptor agonists in adults with overweight or obesity, with particular emphasis on nutrition-related outcomes, dietary intake, gastrointestinal adverse effects, body composition, nutritional adequacy, lifestyle intervention, and multidisciplinary obesity management. Exclusion criteria included studies that focused on outcomes in adults with type 2 diabetes, pediatric populations, non-English-language publications, and articles lacking full-text availability.

Mechanisms of Action for GLP-1s and GIP/GLP-1 RAs

**Figure 1:**
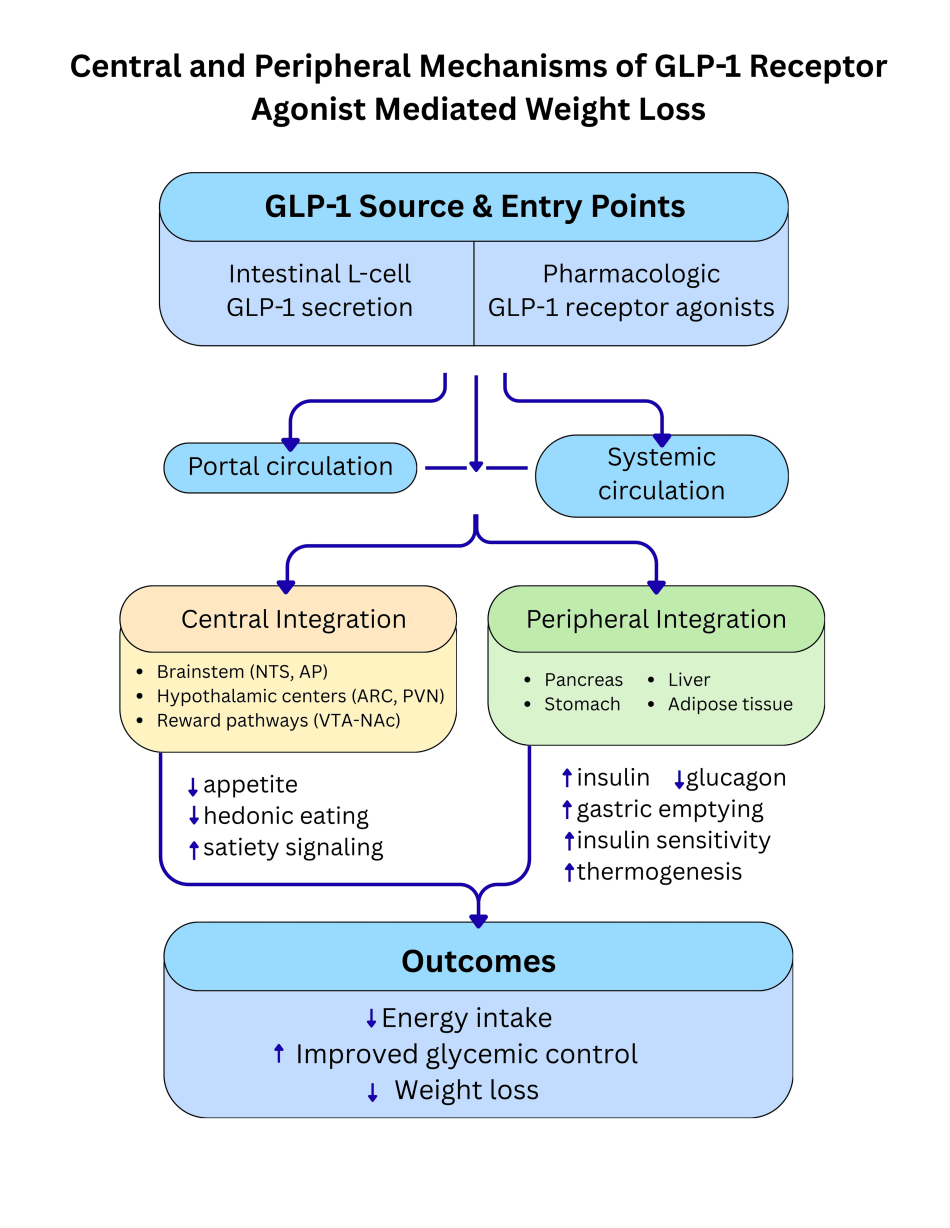
Conceptual model illustrating central and peripheral mechanisms by which glucagon-like peptide-one (GLP-1) receptor agonists influence appetite regulation, glycemic control, and energy balance GLP-1 signaling occurs via vagal pathways and systemic circulation, integrating at the central nervous system and peripheral metabolic targets to reduce food intake and promote weight loss. Concept adapted with permission from [24]. GLP: Glucagon-like peptide.

Although the human body naturally produces glucagon-like peptide-1 (GLP-1) hormones, the half-life of endogenous GLP-1 is only one to two minutes as a result of rapid degradation by the enzyme dipeptidyl peptidase-4 (DPP-4) and renal clearance [22]. GLP-1 RA and GIP/GLP-1 RA drugs are different from endogenous GLP-1 in that they are structurally modified (fatty-acid side chains, albumin binding) to resist DPP-4 degradation, which results in stronger effects and substantially prolonged half-lives that enable once-daily (liraglutide) or once-weekly (semaglutide, tirzepatide) dosing [23]. The drugs are also administered through subcutaneous injection to bypass the gastrointestinal tract, thus allowing them to reach systemic circulation intact to exert their effects. Once in the body, GLP-1 RAs and GIP/GLP-1 RAs may impact weight loss through both central and peripheral pathways. Within the central nervous system, these drugs modulate brain regions (including the hypothalamus and brainstem) that are responsible for controlling appetite, regulating hunger, and energy as they impact neurotransmitter and peptide release [24]. These modulations lead to reduced hunger signals and increased satiety signals that result in a reduction of food intake, a primary driver of weight loss [24]. As GLP-1 RAs bind to GLP-1 receptors located in the nucleus tractus solitarii of the brainstem, serotonergic neuron activity becomes enhanced, promoting satiety and reducing the urge to engage in nonhomeostatic (hedonic) feeding [24]. GLP-1s also activate GLP-1 receptors in several areas of the hypothalamus, leading to an increased release of anorexigenic peptides, decreased release of orexigenic peptides, and a release of corticotropin-releasing hormone, oxytocin, and thyrotropin-releasing hormone that may also lead to a reduction of food intake [24]. Figure 1 below highlights the proposed route of action of GLP-1s in the central and peripheral regulation of feeding and glucose metabolism.

GLP-1s influence weight reduction through delayed gastric emptying, increased satiety, and additional metabolic effects, including improved glycemic control and gut hormone regulation [24]. By delaying the rate at which the stomach empties its contents into the small intestine, GLP-1s result in prolonged feelings of postprandial fullness after consuming food, less frequent hunger, reduced snacking, and smaller meal sizes [24]. These results ultimately manifest in a lower energy intake that may lead to weight loss. When GLP-1 RAs activate GLP-1 receptors on enteric neurons within the gastrointestinal tract, particularly the myenteric neurons, neuronal pathways involving nitric oxide and cyclic adenosine monophosphate (cAMP) are activated, resulting in the relaxation of gastrointestinal smooth muscle (reduced antral and duodenal contractility) and suppression of postprandial motility patterns [25]. At the same time, GLP-1 RAs bind to GLP-1 receptors located on vagal afferent neurons and in the nucleus tractus solitarius (NTS) of the brainstem, which activates the vago-vagal reflex that inhibits gastric motility and increases pyloric tone, consequently slowing the transit of food from the stomach to the duodenum [26]. The quality of data collected relating to the effect of GLP-1s on the rate of gastric emptying is somewhat inconsistent due to the use of measuring techniques such as paracetamol (acetaminophen) absorption instead of the gold standard scintigraphy [27]. A systematic review and meta-analysis conducted by Hiramoto B et al. on the quantitative measures of gastric emptying observed among 15 applicable GLP-1 studies revealed that in the five studies that used scintigraphy, the mean gastric emptying half-life was ≈ 138.4 minutes in the GLP-1 RA arms vs ≈ 95.0 minutes in placebo arms (pooled mean difference of 36 minutes), whereas the 10 studies using acetaminophen showed no statistically significant delay [28]. While studies suggested that the delayed gastric emptying associated with GLP-1s may diminish over time due to tachyphylaxis [28-30] how this impacts the overall weight reduction effects of the drug over time is not yet known. 

In support of weight reduction, GLP-1s may also play roles in modulating gut hormones such as ghrelin, peptide YY (PYY), and cholecystokinin (CCK), all of which have appetite-suppressing effects in the body [24]. GLP-1 receptor activation can blunt the hunger-stimulating effects of ghrelin [31], and although some studies have shown that GLP-1s increase fasting and postprandial peptide PYY [32], others have suggested that long-term use may reduce its secretion through feedback inhibition in humans [33]. While evidence suggesting GLP-1s may increase CCK is limited, one study has shown GLP-1 drugs suppress CCK secretion, leading to reduced gallbladder contractility and may be the contributing factor to the increased risk of gallbladder-related adverse events, such as cholelithiasis and cholecystitis that have been observed with GLP-1 use [34].

An additional component of the GLP-1 RA mechanism of action related to weight loss is the action on pancreatic β-cells to enhance glucose-dependent insulin secretion and suppress glucagon release, which improves glycemic control and reduces lipogenesis [24]. Stable glucose levels support a reduction in appetite that leads to reduced food intake. GLP-1s may reduce triglycerides and LDL cholesterol; however, evidence suggests these improvements are modest [35]. Results from one study using mice observed that GLP-1s stimulated brown adipose tissue (BAT) thermogenesis that was independent of nutrient intake, potentially supporting the weight loss mechanism as well [36]. Research has also shown that GLP-1s reduce pro-inflammatory cytokine production, attenuating oxidative stress and reducing inflammation [37].

GLP-1s also exert hepatic effects by modulating lipid and glucose metabolism [38]. GLP-1s decrease de novo lipogenesis and promote fatty acid oxidation, which results in reduced hepatic fat accumulation [38]. In addition to the organs and systems implicated in the weight-reduction effects of GLP-1s, metabolic actions of these drugs extend into the cardiovascular system, muscles, kidneys, bones, and reproductive systems. For the scope of this review, mechanisms directly related to weight management have been highlighted.

While the mechanisms noted above reflect the actions of GLP-1s (liraglutide, semaglutide), there are also complementary effects in the dual-agonist GIP/GLP-1 (tirzepatide), resulting in additive or synergistic effects on insulin secretion, glucagon regulation, adipose tissue metabolism, and weight loss [39]. GIP/GLP-1 receptor agonists activate both the GLP-1 receptors and the GIP receptors, further enhancing glucose-dependent insulin secretion [40]. GIP receptors also play distinct roles in adipose tissue, such as promoting lipogenesis and modulating lipid metabolism [40]. GIP receptor activation also has a glucagonotropic effect during hypoglycemia, resulting in superior glycemic control and weight loss, compared to GLP-1 RAs alone [39]. 

Weight Loss Observed with GLP-1 and GIP/GLP-1 RA Therapy

In the randomized controlled trials (RCTs) of GLP-1 RAs and GIP/GLP-1 RAs for weight loss in obese/overweight individuals with at least one weight-related comorbidity, the observed mean weight loss has ranged from approximately 5% to over 20% of baseline body weight, depending on the treatment (liraglutide, semaglutide, or tirzepatide), the dose, and the duration of therapy [40]. Table 2 outlines the major randomized controlled trial findings for all three GLP-1 RAs and GIP/GLP-1 Ras, including their associated weight reduction outcomes [41-46]. To date, GLP-1 RAs and GIP/GLP-1 RAs are the pharmaceutical drug class that achieves the greatest magnitude of weight loss, with phentermine/topiramate a close second to liraglutide [47].

**Table 2:** Summary of results from the pharmaceutical company-funded RCTs GLP: glucagon-like peptide, GIP: glucose-dependent insulinotropic polypeptide, SCALE: Satiety and Clinical Adiposity—Liraglutide Evidence, BMI: body mass index, STEP: Semaglutide Treatment Effect in People with Obesity.

GLP-1, GIP/GLP-1		Trial	Published	Design & population	Duration	Main Weight Reduction Outcome
Liraglutide (Saxenda, 3.0 mg daily)		SCALE Obesity & Prediabetes [14]	2015	randomized, double-blind, placebo-controlled trial of adults with obesity of at least 30 or a BMI of at least 27 with one comorbidity	56 wk + 2-yr extension	−8.0% mean loss vs −2.6% placebo
		SCALE Maintenance [41]	2013	randomized, double-blind, placebo-controlled trial of adults with obesity of at least 30 or a BMI of at least 27 with one comorbidity who lost ≥5% of initial weight during a LCD run-in	56 wk	Initial weight loss was maintained and 6.2% additional loss vs 0.2% on placebo
Semaglutide (Wegovy, 2.4 mg weekly)		STEP 1 [16]	2021	randomized, double-blind, placebo-controlled trial of adults with obesity of at least 30 or a BMI of at least 27 with one comorbidity	68 wk s	−14.9 % mean loss vs −2.4% placebo
		STEP 3 [42]	2021	randomized, double-blind, parallel-group placebo-controlled trial of adults with obesity of at least 30 or a BMI of at least 27 with one comorbidity as an adjunct to intensive behavioral therapy	68 wks	–16.0% mean loss vs –5.7% for placebo
		STEP 4 [43]	2021	randomized, double-blind, withdrawal study of adults with obesity of at least 30 or a BMI of at least 27 with one comorbidity. 20 wk open-label run-in on semaglutide 2.4 mg, then re-randomized to continue semaglutide vs switch to placebo	68 wk (48 wk randomized phase)	Those continuing semaglutide maintained/continued losing (−7.9%), while those switched to placebo regained ~6.9% of body weight
		STEP 5 [44]	2022	randomized, double-blind, placebo-controlled trial of adults with obesity of at least 30 or a BMI of at least 27 with one comorbidity	104 wks	−15.2% vs −2.6% placebo
Tirzepatide (Zepbound; 5, 10, 15 mg weekly)		SURMOUNT-1 [19]	2022	randomized, double-blind, placebo-controlled trial of adults with obesity of at least 30 or a BMI of at least 27 with one comorbidity	72 wks	-15.0% with 5-mg weekly doses of tirzepatide, -19.5% with 10-mg doses, and -20.9% with 15-mg doses versus -3.1% with placebo
		SURMOUNT-3 [45]	2023	randomized, double-blind, placebo-controlled trial of adults with obesity of at least 30 or a BMI of at least 27 with one comorbidity who had already lost ≥5% weight following a 12‐week intensive lifestyle program	72 wks	Additional −18.4% vs +2.5% placebo
		SURMOUNT-4 [46]	2024	randomized, double-blind, placebo-controlled trial of adults with obesity of at least 30 or a BMI of at least 27 with one comorbidity; 36-week, open-label tirzepatide lead-in period followed by a 52-week, double-blind, placebo-controlled period	88 wks	Continue group maintained −25.3% total loss; placebo group regained ~14% weight

A systematic review of 26 randomized controlled trials by Moiz A et al. [48], including over 15,000 participants, reported that tirzepatide (15 mg once weekly) resulted in weight loss of up to 17.8% after 72 weeks of treatment, semaglutide (2.4 mg once weekly) up to 13.9% after 68 weeks of treatment, and liraglutide (3.0 mg once daily) up to 5.8%. As with all AOMs, GLP-1 RAs and GIP/GLP-1 RAs are associated with a variety of adverse events.

Adverse Events Associated with GLP-1 RAs and GIP/GLP-1 RAs

GLP-1 RA and GIP/GLP-1 RA use is often accompanied by a range of adverse side effects that can impact patient adherence and overall tolerability. Gastrointestinal disturbances, including nausea, vomiting, diarrhea, and delayed gastric emptying, are among the most commonly reported issues. While generally considered safe, these adverse effects can vary in severity and duration, underscoring the importance of careful monitoring, patient education, and individualized management strategies to optimize treatment outcomes. Compared to other weight loss pharmacotherapies, GLP-1 RAs tend to have a higher incidence of gastrointestinal side effects, whereas medications like orlistat primarily cause fat-malabsorption-related issues such as oily stools and flatulence, and newer agents like bupropion/naltrexone are more often associated with neuropsychiatric or cardiovascular effects [21].

The most common side effects associated with GLP-1 RAs and GIP/GLP-1 RAs, as noted on their respective FDA labels, are shown in Table 3. The main black box warning for GLP-1 agonists is the risk of thyroid C-cell tumors, including medullary thyroid carcinoma (MTC) [15,17,20].

**Table 3:** Adverse reactions noted on FDA labels for GLP-1 RAs and GIP/GLP-1 RAs FDA: Food and Drug Administration, GLP: glucagon-like peptide, GIP: glucose-dependent insulinotropic polypeptide, RA: receptor agonists.

GLP-1, GIP/GLP-1		Adverse reactions noted on the FDA label
Liraglutide [15](Saxenda, 3.0 mg daily)		Most common adverse reactions, reported in greater than or equal to 5% are: nausea, hypoglycemia, diarrhea, constipation, vomiting, headache, decreased appetite, dyspepsia, fatigue, dizziness, abdominal pain, and increased lipase
			Semaglutide [17] (Wegovy, 2.4 mg weekly)		The most common adverse reactions, reported in greater than or equal to 5% of patients treated with Wegovy, are: nausea, diarrhea, vomiting, constipation, abdominal pain, headache, fatigue, dyspepsia, dizziness, abdominal distension, eructation, hypoglycemia in patients with type 2 diabetes, flatulence, gastroenteritis, and gastroesophageal reflux disease
Tirzepatide [20] (Zepbound; 5, 10, 15 mg weekly)		The most common adverse reactions, reported in ≥5% of patients treated with Zepbound, are: nausea, diarrhea, vomiting, constipation, abdominal pain, dyspepsia, injection site reactions, fatigue, hypersensitivity reactions, eructation, hair loss, and gastroesophageal reflux disease			

The most common adverse side effects associated with the use of the three major GLP-1 drugs are nausea, diarrhea, vomiting, and constipation, all reportedly mild to moderate in nature, usually occurring within the first 48 hours after drug initiation, and after each dose increase [49]. These are among the many concerns that require attention and support, given their implications for a patient’s overall well-being, continuation of treatment, nutrient status, and quality of life. In a comprehensive clinical review of several GLP-1 studies, Ghusn et al. outlined the most commonly reported side effects. Consistent with this compilation, analysis of the U.S. FDA Adverse Event Reporting System (FAERS) database similarly identifies gastrointestinal disorders, particularly nausea, vomiting, and diarrhea, as the most common adverse events associated with GLP-1 RAs and GIP/GLP-1 RAs [50]. Studies have also detected cases of acute pancreatitis, acute gallbladder disease, and several other significant adverse events, such as osteomyelitis, renal cell carcinoma, nephrolithiasis, and drug-induced liver injury [49,50]. A recently published review analyzed a myriad of recent investigations and presented possible links between GLP-1 RAs and additional side effects, such as intestinal obstruction, aspiration occurring during procedural anesthesia, ocular adverse effects (diabetic retinopathy), various neoplasms (MTC, breast cancer, and cholangiocarcinoma), and sarcopenia [51]. Data on adverse events have substantive limitations. Reviews of multiple pharmacovigilance studies highlighted that spontaneous reporting systems were subject to significant underreporting, reporting bias, and confounding, which limit the ability to accurately estimate the true incidence of adverse events [52]. * *

Gastrointestinal side effects represent the most frequently reported adverse events for both semaglutide and tirzepatide, with approximately 75% of individuals receiving either injectable or oral semaglutide experiencing gastrointestinal symptoms, compared to 30-40% for tirzepatide [53].

Another consequence of GLP-1 RA use that is of major concern is the possible loss of lean muscle mass, although studies vary in their assessment of just how much. A systematic review and meta‐analysis assessing the effects of GLP-1 RAs on various measures of muscle mass in individuals living with overweight or obesity concluded that reductions in muscle mass accounted for less than 20% of the total weight reduction experienced by patients [54]. Based on their findings, the authors concluded that the proportion of muscle mass lost to total weight loss with respect to GLP-1 RAs may actually be less than that observed in bariatric surgery patients and those losing weight due to dietary interventions [54]. Of the 46 studies included in this review, only 22 utilized the gold standard dual-energy x-ray absorptiometry (DEXA) scan to evaluate body composition, indicating a need for additional research. A commonly cited rule of thumb in the body-composition/weight-loss literature states that approximately 25% of total weight loss ends up being fat-free mass (FFM) that includes lean tissue, skeletal muscle, and bone [55]. The percentage may be slightly less for females. A recent study showed that in voluntary caloric restriction in nonelderly adults with overweight or obesity, approximately 2 to 2.5 kg of skeletal muscle mass is lost per 10 kg of total body weight reduction in males, and about 1 to 1.5 kg per 10 kg in females when no structured exercise program is implemented [56]. A review of 38 studies involving patients with and without T2DM concluded that loss of lean tissue accounted for less than 20% of the total weight loss, with fat mass reduction being much greater [57]. Another systematic review and meta-analysis, however, observed reductions in lean mass ranging as high as 40% to 60% as a proportion of total weight lost [58]. Despite variance in overall percentages, loss of FFM is an area that warrants targeted mitigation strategies to preserve lean tissue and prevent the associated harm (increased morbidity and mortality, reduced quality of life, functional decline) that comes with its decline, including evidence-based nutrition intervention, supplementation, and weight-bearing exercise.

While glucagon-like peptide-1 receptor agonists (GLP-1 RAs) and glucose-dependent insulinotropic polypeptide/glucagon-like peptide-1 receptor agonists (GIP/GLP-1 RAs) are associated with well-documented adverse events, particularly gastrointestinal effects, emerging research demonstrates potential benefits extending beyond weight loss and glycemic control [59,60]. These agents have shown promise in cardiovascular protection, with reductions in major adverse cardiovascular events and heart failure hospitalizations, as well as renal benefits including slowed decline in estimated glomerular filtration rate and reduced albuminuria [59,61,62]. In addition, novel therapeutic applications are being explored in conditions such as metabolic dysfunction-associated steatohepatitis, obstructive sleep apnea, knee osteoarthritis, neurodegenerative diseases including Alzheimer's and Parkinson's disease, and substance use disorders [59,60,62,63]. Some of these benefits appear to be independent of weight loss or glucose control, potentially reflecting direct anti-inflammatory effects and actions on the heart, liver, blood vessels, and central nervous system [60,63,64].

Nutrition-Related Outcomes Observed with GLP-1s and GIP/GLP-1s

Although the existing literature provides a sizable amount of data regarding the side effects, adverse events, and weight loss observed in GLP-1 treatment for obesity, one area of understanding where a large knowledge gap exists is in the nutrition-related outcomes. Nutrition-related outcomes deserve a special focus given their clinical implications for the long-term health and quality of life for patients being treated with GLP-1s. An extensive understanding of the quality and quantity of calories consumed by patients on GLP-1 RAs, as well as any nutrient deficiencies that may result from treatment, has not been fully elucidated in the current literature. A small cross-sectional study [65] was recently conducted with the aim of exploring nutrient intake; however, the small participant size, limited treatment time, and three-day food records provided limited findings.

In a cross-sectional study, Johnson et al. [65] concluded that patients with obesity using GLP-1 RAs commonly have dietary intakes below the Dietary Reference Intakes (DRI) for several essential nutrients, including fiber, calcium, iron, magnesium, potassium, choline, vitamin A, vitamin C, vitamin D, and vitamin E. Additionally, the patients tended to consume a higher percentage of calories from fat and saturated fat than is recommended [65]. Protein intake, although within the acceptable macronutrient distribution range (AMDR) as a percentage of total calories, was significantly below daily needs when assessed by grams per kilogram per day, which is particularly relevant during weight loss [65]. Interestingly, participants met DRIs for most B-vitamins, copper, phosphorus, selenium, and zinc [65]. Given the limitations of the study, including a lack of baseline biomarker assessments and a longitudinal follow-up to assess how nutrient intake changes over time, their findings are relatively inconclusive in nature. Three-day food records are highly susceptible to under-reporting, recall bias, and reactivity; participants often alter their intake due to awareness of being monitored. The limitations severely compromise the internal validity and accuracy of reported dietary intake, making it challenging to draw any valid conclusions regarding intake patterns and resulting nutrient deficiencies. Nonetheless, the study was a pivotal step in the direction of fulfilling the need for further studies of this kind and highlights the importance of nutrition support during GLP-1 treatment. Nutrient deficiencies that result from the GLP-1 and GIP/GLP-1 RA treatments may be clinically significant, as they can directly influence metabolic health, treatment tolerance, and long-term outcomes. The medications change how people eat, how much they eat, and how nutrients are absorbed and utilized, creating measurable physiological consequences. Baseline and ongoing CBC, ferritin ± iron studies, B12, folate, 25-OH-D, calcium, CMP, albumin, magnesium, and functional nutrient testing are recommended to assess the impact of GLP-1s on overall health.

A larger, observational, retrospective analysis of data from 461,382 adults who underwent GLP-1 treatment between 2017 and 2021 with no prior diagnoses of nutritional deficiencies was conducted by Butsch et al. [66], although most patients not only had obesity, but had type 2 diabetes (T2DM) as well (≈ 80.5 %). It should be noted that most participants in the study were concurrently on Metformin, which has itself been linked to nutrient deficiencies, specifically vitamin B12 [67,68]. Despite notable limitations, the study observed findings consistent with the notion that GLP-1 RA treatment may lead to or exacerbate nutrient deficiencies. This study represents one of the few large-scale analyses available that investigates nutrient deficiencies in a real-world GLP-1 RA-treated population. Assorted nutrient deficiencies were diagnosed in 12.7 % of the patients within six months of treatment initiation, escalating to 22.4% within a year [66]. Vitamin D deficiency was the most common deficiency identified, having an occurrence of 7.5 % and 13.6 % within six and 12 months, respectively [66]. Additionally, nutritional anemia was the most common deficiency-related complication identified among participants (2.1% after six months, 4% after one year) [66]. Although the analysis sheds a slightly stronger light on some of the possible nutrient deficiencies associated with GLP-1 use, a lack of data on dietary intake, supplement use, physical activity, or body composition changes (beyond claims) continues to exist. The lack of baseline and longitudinal data on dietary intake and supplement use directly prevents attributing observed nutrient deficiencies solely to GLP-1 use. Given that the study was observational, several additional confounding factors cannot be fully excluded, and causation cannot be definitively established. Furthermore, the study did not include tirzepatide (not FDA approved at the time). To fill in the critical knowledge gaps, prospective study designs are needed to rigorously collect baseline and longitudinal data on all confounding factors and include validated and detailed dietary assessments, comprehensive supplement logs, and objective measures of physical activity.

Beyond the highly limited studies just noted, detailed data on the nutrient-related deficiencies that may result from GLP-1 RA treatment for obesity remain unidentified. Several studies do, however, provide some small insights into how the drugs impact other facets of dietary consumption. Christensen et al. [69] analyzed ten studies from various countries that included patients on different GLP-1 RA treatments. Many of the studies involved patients who presented with obesity and T2DM and compiled data regarding overall energy intake reductions observed. Overall, the authors report that total energy intake was reduced by 16-39% across studies, with very few of them evaluating diet composition [69]. The possibility for such a large reduction in energy intake during treatment may lead to a subsequent reduction of intake in key macro and micronutrients. Evidence from clinical trials and observational data indicates that during the early phases of GLP-1 treatment, some individuals may reduce their energy intake to below 800 kcal per day [70]. However, Quast et al. [71] pooled data showing that the GLP-1 treatment group exhibited a roughly ~17% reduction in protein intake, ~22.7% reduction in fat intake, and ~12.2% reduction in carbohydrate intake. However, the applicability of these findings is limited, as the study took place in Germany, where dietary patterns are different than those in the United States. Overall, the current literature provides limited data on the specific composition of dietary intake in GLP-1 treatment, as most focus on quantifying total caloric reduction rather than characterizing changes in diet quality or nutritional adequacy.

A small number of studies report participants’ insights on food cravings and preferences as impacted by GLP-1 RA treatment. In a secondary analysis of the SURMOUNT-1 [19] trial with patients treating obesity with tirzepatide, significant reductions in overall food cravings and in preference for high-carbohydrate and high-fat foods were self-reported by participants [71]. A correlation was noted between the reduction in overall food-craving scores and the overall weight reduction in the treatment group, suggesting that decreased cravings may contribute to the medication’s weight‑loss mechanism [72]. The relatively small treatment group in the study had undergone counselling to support a low-calorie diet, so the observations reported may not be representative of the greater population on a tirzepatide protocol.

Martin et al. [73] conducted a six-week phase one study aimed at investigating the initial effects of tirzepatide on energy intake, calculating an estimated treatment difference of −524.6 kcal daily compared to placebo. As evidenced by self-reported measures in the study, tirzepatide significantly reduced overall appetite, hunger, and cravings for high-fat, sweets, and carbohydrate/starch foods while decreasing reactivity to food cues in the environment and the tendency to overeat [73]. A reduction of roughly 525 kcal/day, if sustained, equates to ≈ a 3,675 kcal/week deficit, or roughly 1 lb (0.45 kg) of fat loss per week; ~2.5-3 kg (5-7 lb) of theoretical weight reduction over six weeks. Studies on liraglutide and semaglutide have reported similar declines in taste preference for sweet, salty, savory, or fatty foods as well as greater control of eating [32,74,75]. Semaglutide studies have observed participants’ mean energy intake during an ad libitum lunch to be 35% less than the placebo, with one study noting a ~47% reduction in intake relative to baseline [75,76].

Support and Intervention Considerations for Nutrition Practitioners

Aside from the quantity of calories consumed, data regarding how GLP-1s affect the timing of meals, or any other aspect of dietary quality, such as macronutrient and micronutrient composition, that could provide the foundation for evidence-based guidance in the efforts to support health, is yet to be uncovered. Patients on GLP-1s require counselling and support to maximize treatment outcomes through strategic nutrition aimed at preventing deficiencies, preserving muscle mass, and managing adverse side effects. As the knowledge gap remains to be addressed, and the need to support patients with obesity on GLP-1 RAs becomes increasingly more apparent, interprofessional collaborative efforts are emerging with the objective to provide nutrition professionals with the most appropriate possible guidance to this end. Expert groups have shed light on the need for additional research while presenting the best available information for clinicians to utilize in patient-centered care. A referral to a registered dietitian nutritionist (RDN) should be a critical component of GLP-1 therapy to ensure safety, manage expectations, provide education, maximize benefits, and promote long-term health. The interdisciplinary panel suggests a multi-faceted approach to GLP-1 patient care that incorporates eight major principles: initiation of GLP-1 therapy with a patient-centered approach, completion of baseline nutritional assessment and screening, management of gastrointestinal side effects, navigation of dietary preferences and intakes, prevention and mitigation of nutrient deficiencies, preservation of muscle and bone mass, maximization of weight reduction efficacy, and promotion of other supportive lifestyle measures [4]. The organizations emphasize that successful outcomes rely on early dietitian involvement, exercise (especially resistance training), and attention to social and behavioral factors to optimize adherence and long-term health, while proactive monitoring of diet quality, body composition, and side effects is essential to maximize the benefits of GLP‑1 RA therapy and minimize risks and support sustainable weight management [4].

Nutrient Deficiencies

As dietitians seek to understand how to best serve patients with obesity on GLP-1 RAs, a starting point for intervention or support is to understand what is known about the usual intakes and dietary habits of individuals with obesity before the start of a GLP-1 treatment. Malnutrition is relatively common among individuals with obesity, creating a paradoxical condition in which excessive energy intake coexists with deficiencies in vital micronutrients due to poor dietary quality, limited access to nutrient-dense foods, impaired nutrient absorption or metabolism, altered distribution or excretion, and systemic inflammation that disrupts micronutrient balance [77]. The most consistently reported deficiencies among individuals with obesity include vitamin D, iron, magnesium, zinc, folate, vitamin B12, and vitamin A, vitamin E, calcium, potassium, and fiber [78,79]. As energy intake is significantly reduced with GLP-1 RAs, malnutrition may be further exacerbated.

The American College of Lifestyle Medicine, the American Society for Nutrition (ASN), the Obesity Medicine Association, and The Obesity Society jointly advise that individuals with obesity on GLP-1 therapy should engage in active management of dietary composition to prevent and address nutrient deficiencies [4]. In-depth baseline nutrition assessment, and all facets of medical nutrition therapy (MNT) provided by RDNs as part of the standards of care for patients with obesity [80] should be mandated as an adjunct to a GLP-1 prescription. Within the MNT framework, RDNs should conduct a comprehensive baseline nutritional assessment, covering current dietary patterns, anthropometrics, body composition via DEXA if possible, and relevant biochemical markers like vitamin D, B12, iron, and albumin, individualized meal planning, and structured education. As energy consumption is reduced, dietary choices become exponentially more significant in the quest to fulfill adequate nutrient intakes, requiring proactive support and professional guidance to meet the unique needs of the patient. The joint advisory from the organizations highlights that structured, frequent RDN involvement may contribute to greater weight loss and improved metabolic outcomes for patients [4].

In addition, the joint advisory from the American College of Lifestyle Medicine, the American Society for Nutrition, the Obesity Medicine Association, and The Obesity Society outlines several key nutritional priorities for patients on GLP-1 therapy for obesity [4]. They recommend adherence to a dietary pattern characterized by a wide variety of nutrient-dense, minimally processed foods, including fruits, vegetables, whole grains, legumes, lean protein sources, nuts, seeds, and plant-derived oils, along with targeted supplementation to prevent or correct deficiencies where appropriate [4]. Furthermore, clinicians are encouraged to counsel patients to adopt dietary patterns that minimize the consumption of ultraprocessed foods, refined carbohydrates, sugar-sweetened beverages, and red and processed meats while emphasizing structured eating routines that include appropriately portioned meals consumed at consistent intervals [4]. Obese individuals have increased levels of inflammation, as evidenced by elevated pro-inflammatory cytokines and immune cell activation [81], and would significantly benefit from consuming anti-inflammatory diets, such as the Mediterranean diet. Restrictive dietary patterns, such as keto or intermittent fasting, should be closely monitored and evaluated to ensure that they are not a catalyst in advancing nutrient deficiencies as well. This is a population that will require substantial support. Education to promote self-efficacy and an understanding of what to consume to prevent nutrient deficiencies is vital. Individuals with obesity, at baseline, present with nutrient deficiencies and inflammation. Malnutrition, although paradoxical, is very common. Although nutrition protocols to support GLP-1 patients, such as anti-inflammatory diets, high protein, and high fiber, exist, the reality is that if it were easy to get patients to adopt those eating patterns, they probably wouldn’t be on a GLP-1 treatment. It takes time to achieve those behavioral modifications. Although the first line of intervention in nutrition care is to pursue a food-first approach to meeting nutrient needs, given the large reduction of calories and often suboptimal baseline eating patterns, supplements will be necessary to meet all the nutrient needs for this population. When consuming significantly reduced calories, nutrient density and supplementation are critical. Particularly in the efforts to preserve lean muscle, protein shakes and supplements that are easy to take will be an essential component of a protocol to support the preservation of lean mass. Protein in and of itself promotes satiety, so individuals on GLP-1s tend to have difficulty trying to take down a steak. RDNs will also need to provide a lot of gut health support to address dysbiosis and intestinal permeability to ensure the nutrients are being properly absorbed. As new dietary patterns are adopted and root causes are addressed, ongoing nutrition assessments may support changes in supplementation. Early on and if not throughout the course of long-term treatments, multivitamins and supplements will be a critical puzzle piece to fill in nutrition gaps. The more we look at the limited, available evidence on GLP1s, the more we see a vital need for dietitians to be an integral part of therapy. 

Preservation of Lean Mass

Preservation of skeletal muscle and bone mass should be prioritized during any weight reduction intervention, including GLP-1 RA treatment. In the absence of a DEXA scan, RDNs may use validated anthropometric equations or bioelectrical impedance analysis (BIA) to estimate lean body mass. Because of hydration variability, geometry differences, and poor population-specific calibration, the tools are less reliable at BMI > 35 and should be interpreted cautiously. The tools can still provide foundational data points that can be used in the creation and monitoring of nutrition interventions for GLP-1 patients. Adequate dietary protein intake, around 1.5 g per kilogram of FFM, in conjunction with regular resistance or strength-based exercise, is recommended to attenuate the loss of lean body and bone mass commonly observed with rapid weight loss [4]. In the absence of hunger, nutrient-dense smoothies or protein drinks may be appropriate to meet nutrient needs [4]. The American Society for Parenteral and Enteral Nutrition’s (ASPEN) practice tool for clinicians cites specific strategies for protein consumption, including prioritizing consuming high-protein foods first within a meal [82]. Adequate consumption of dietary fiber (> 21 g/d for women, > 30 g/d for men), including in supplement forms, is also emphasized to support health and alleviate constipation, although intake amounts may need to be increased slowly, especially during the initial weeks of GLP-1 treatment when adverse side effects tend to peak [4]. Physical activity and weight-bearing exercise, as appropriate, should be emphasized for their role in supporting maintenance and growth of lean muscle, especially since rapid weight loss is associated with loss of lean mass. Special considerations should be taken with adults with obesity, so a referral to a certified personal trainer who can provide client-centered modifications is warranted. Often, adults with obesity struggle to exercise because of a mix of physical discomfort, low confidence, and experiences of stigma, combined with limited access to supportive, size-inclusive environments. For adults with obesity, resistance training should prioritize 2-3 sessions per week, multi-joint exercises, and moderate loads (60-80% 1-Rep Max) to build strength and preserve lean mass while using controlled tempo, appropriate rest, and accessible equipment to reduce joint stress and improve adherence. The key is to maintain adequate weekly volume (≥6-10 sets per muscle group) and progress gradually to safely improve metabolic health, function, and body composition.

Managing Adverse Events

Although numbers vary across studies, discontinuation due to adverse events is estimated at around 10% for all three GLP-1s [53], further highlighting the need for patient support by dietitians to navigate the challenges of treatment. Proactive management of side effects through education and managing expectations is considered crucial for maintaining engagement and minimizing discontinuation of GLP-1s. Ongoing monitoring and individualized counseling by RDNs is recommended to tailor dietary patterns, assess for deficiencies, and adjust recommendations based on treatment response and patient preferences. Especially during the initial weeks of treatment, extra consideration should be taken to mitigate gastrointestinal side effects associated with GLP-1s. Adequate hydration should always be an area of priority, and possible dehydration resulting from any vomiting in early treatment stages or from experiencing early satiety should be addressed. Patients should also be advised on protocols to reduce nausea, such as ginger, teas, or eating slowly, avoiding strong smells, eating a bland diet, and avoiding high-fat, fried foods. It is recommended that at least 30 minutes have passed since the most recent GLP-1 RA dose before patients may consume foods that help alleviate nausea [83]. Short-term use of antiemetic agents, such as ondansetron, may be prescribed by physicians in cases of significant or severe nausea and vomiting [84]. Delayed intestinal motility associated with GLP-1 RA use may also warrant proactive management with bulk-forming agents, such as psyllium husk, as well as stool softeners, and osmotic or stimulant laxatives to promote motility and passage [84]. In the event of gastroesophageal reflux, advising smaller portions, limiting high-fat or spicy foods, and remaining in an upright position for the first two to three hours post-meal should be recommended [85]. Although no high-quality randomized controlled trials or expert consensus statements support the use of probiotics for GLP-1 RA-related GI symptoms, they may be reasonable to employ in the efforts to maintain microbial diversity, support gut barrier integrity, and correct the dysbiosis associated with obesity [86]. GLP-1 RA drugs modestly reshape the gut microbiota through a combination of direct drug effects, slowed gastric emptying, and weight-loss-related change, but the specific patterns and clinical significance are still being clarified and are likely very patient specific, given the impact of diet. A systematic review highlights that the effects of GLP-1 analogues on the gut microbiota are agent-specific and influenced by host factors and study design [87]. The clinical implications of microbiota changes remain uncertain, and further research is needed to clarify their impact on patient outcomes. Diarrhea may be addressed with generous hydration and temporary avoidance of dairy, high fiber, coffee, and products with sweeteners ending in “ol,” such as sorbitol, mannitol, xylitol, maltitol, including candy and gum [80].

Weight Regain

In a narrative review of 13 RCTs, discontinuation of GLP-1s in adults treated for obesity was observed to lead to rapid weight regain, regardless of the duration of prior therapy [88]. Although the reasons for discontinuation may be nuanced and varied, the phenomenon further highlights the need for nutrition practitioners to support patients through treatment challenges while educating them on how to adopt the eating habits and lifestyle modifications that may facilitate maintenance of weight loss despite GLP-1 cessation. Developing structured, long-term maintenance programs should include sustained behavioral therapy, personalized meal planning, and regular follow-ups to provide concrete, implementable interventions to maintain the impact of GLP-1s. A focus on behavior modification and skill-building is foundational in overcoming the challenge to prevent weight regain if GLP-1 treatment is to end. A systematic review and meta-analysis that included eight RCTs with 2,372 participants showed that weight regain for GLP-1 users was proportional to the initial weight lost [89]. Patients who discontinued liraglutide regained a mean of 2.20 kg while those who discontinued semaglutide or tirzepatide regained a mean of 9.69 kg [89]. Evidence indicates that discontinuing semaglutide or tirzepatide therapy leads to significant weight regain and a reversal of the metabolic improvements previously achieved, including those in HbA1c and lipid levels [53]. GLP-1 RAs may best be viewed as chronic, long-term treatments, similar to antihypertensive or lipid-lowering medications as opposed to short-term weight-loss aids. Therefore, sustained lifestyle, behavioral, and nutritional intervention and ongoing support are essential for maintaining weight reduction if pharmacotherapy stops.

Education and Lifestyle Modifications

Because of their efficacy in weight-reduction outcomes, GLP-1s can often lead patients to misconceive that the drugs are a suitable replacement for healthy eating choices and regular physical activity. To optimize health and weight loss outcomes and support long-term sustainability, encouraging lifestyle modifications and education are an essential part of patient care by a nutrition practitioner. Sleep hygiene, stress management, and social support should also be included in the multifaceted treatment support plan. Some dietitians report that using visual and metaphorical aids enhances patient understanding of medication effects and dietary recommendations [90]. RDNs can improve adherence to GLP-1 therapy by using motivational interviewing, symptom-focused nutrition strategies, simple meal frameworks, and highly practical habit-based goals. Frequent support, anticipatory guidance, and side-effect management assist patients in sustaining lifestyle changes while minimizing pharmacotherapy dropout.

Structured Framework for Dietitians using the NCP (Nutrition Care Process)

**Figure 2:**
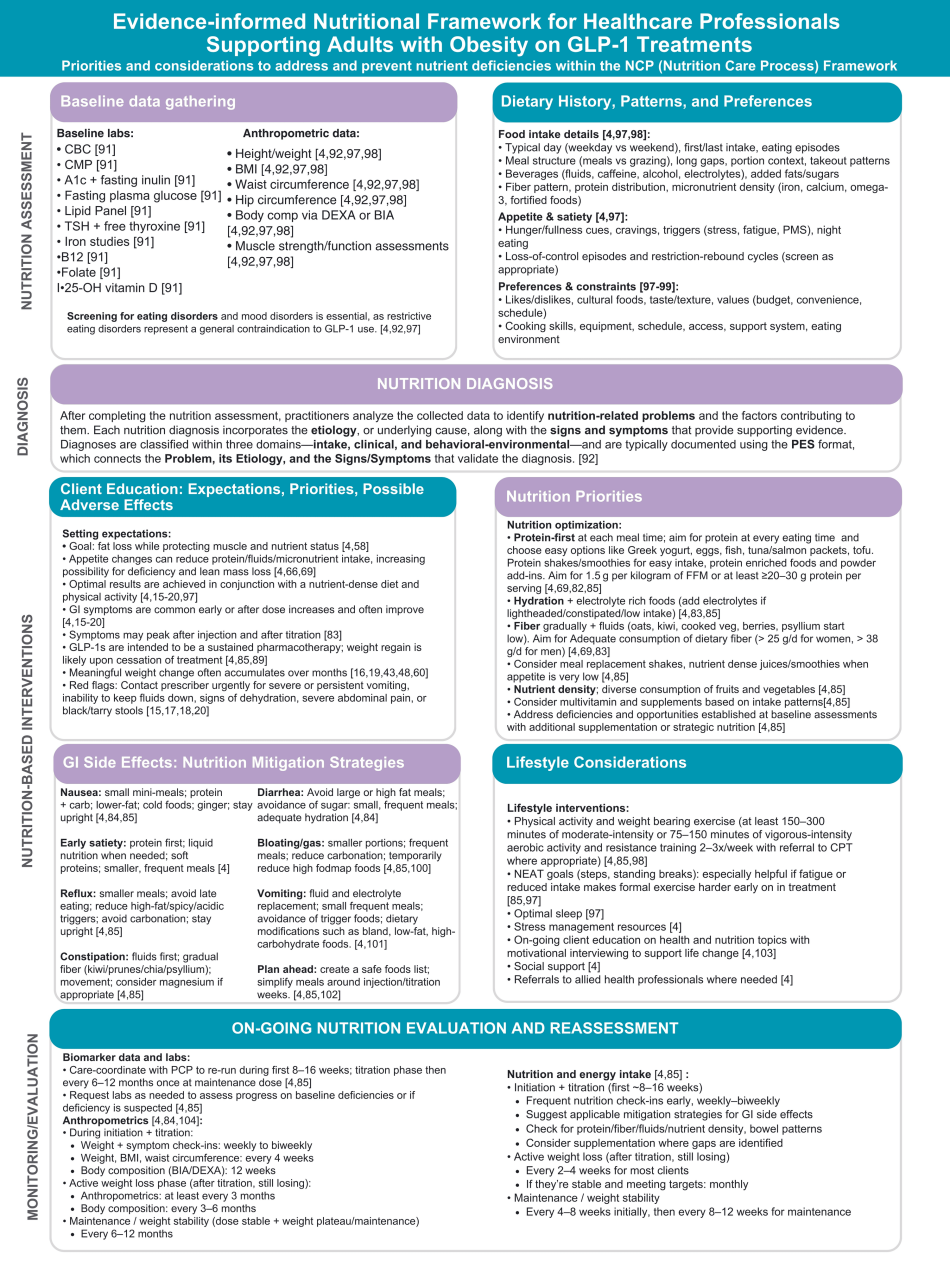
Evidence informed nutritional framework for healthcare professional supporting adult with obesity on GLP-1 treatment CBC: complete blood count, CMP: comprehensive metabolic panel, TSH: thyroid-stimulating hormone, OH-D: hydroxyvitamin D, BMI: body mass index, DEXA: dual-energy x-ray absorptiometry, BIA: bioelectrical impedance analysis, CPT: certified personal trainer, PCP: primary care physician, NEAT: non activity exercise thermogenesis. McGuane, C. Own work created with CANVA.

Given the appetite suppression, delayed gastric emptying, gastrointestinal adverse effects, and potential for reduced overall nutrient intake associated with glucagon-like peptide-1 receptor agonists (GLP-1 RAs) and dual GIP/GLP-1 receptor agonists, a structured and proactive nutrition strategy is essential to mitigate unintended nutritional compromise while optimizing therapeutic outcomes. Although these pharmacotherapies demonstrate robust efficacy for weight reduction and cardiometabolic improvement, emerging clinical guidance emphasizes the importance of preserving lean mass, preventing micronutrient deficiencies, and addressing gastrointestinal intolerance during both initiation and maintenance phases of treatment. The practical framework presented in Figure 2 integrates the Academy of Nutrition and Dietetics’ Nutrition Care Process (NCP) with GLP-1-specific considerations, organizing care into four domains: comprehensive baseline assessment (including dietary patterns, anthropometrics, and relevant laboratory biomarkers), individualized nutrition diagnosis using PES statements, targeted nutrition interventions (protein-forward dietary strategies, hydration and fiber optimization, GI symptom mitigation, and lifestyle integration), and structured ongoing monitoring and reassessment [91-104]. This framework further incorporates anticipatory guidance on appetite suppression, peak symptoms during titration, and the chronic nature of GLP-1 pharmacotherapy, noting the risk of weight gain after discontinuation. By integrating pharmacologic obesity treatment with systematic medical nutrition therapy in line with current literature and clinical guidance, this model provides healthcare professionals with a practical framework to support nutrient adequacy, symptom management, sustainable behavior, and long-term metabolic health for adults on GLP-1-based therapies.

 Discussion

The findings of this literature review highlight that although glucagon-like peptide-1 receptor agonists (GLP-1 RAs) and dual glucose-dependent insulinotropic polypeptide/glucagon-like peptide-1 receptor agonists (GIP/GLP-1 RAs) represent a remarkable breakthrough in obesity pharmacotherapy, their rapid adoption has outpaced the development of nutritional frameworks to support patients. The literature consistently confirms substantial weight loss outcomes, ranging from approximately 5% to over 20% of baseline body weight [40] and highlights critical uncertainties regarding their long-term nutritional and metabolic consequences. The scarcity of data on dietary quality, micronutrient adequacy, and body composition outcomes emphasizes a gap in clinical knowledge that warrants urgent attention from researchers so that evidence-based guidance for nutrition practitioners may be devised.

Interdisciplinary collaboration is needed to support the optimal outcomes and long-term health of patients with obesity on GLP-1 RA treatments, including care coordination with physicians and pharmacists. Interdisciplinary collaboration is essential for patients with obesity on GLP-1 RA therapy because no single discipline can fully address all the metabolic, nutritional, behavioral, and medical complexities the patients face. Physicians manage GLP-1 selection, titration, and safety; RDNs optimize nutrient intake, mitigate side effects, and preserve lean mass; behavioral health providers support eating-behavior change and emotional regulation; and personal trainers ensure safe, progressive activity to maintain function and metabolic health. When allied health professionals communicate and coordinate care, patients receive a more comprehensive, sustainable plan, ultimately leading to better adherence, fewer complications, and stronger long-term outcomes.

Integration of Current Evidence

Across various studies, GLP-1 RA and GIP/GLP-1 RA therapies produce weight loss magnitudes previously unattainable with earlier pharmacologic options. However, the mechanisms that mediate the effects, such as appetite suppression, delayed gastric emptying, and altered gastrointestinal signaling, may simultaneously create nutritional vulnerabilities that must be addressed. Significant caloric reductions of intake as low as less than 800 kcal per day in early treatment may predispose patients to suboptimal macro- and micronutrient consumption or exacerbate existing deficiencies.

Clinical Implications for Nutrition Practice

Given the nutrition-related concerns, the role of registered dietitian nutritionists (RDNs) and nutrition practitioners becomes indispensable. Integrating individualized medical nutrition therapy (MNT) early in the treatment process can mitigate adverse outcomes, improve tolerability, and enhance long-term success. Current interprofessional advisories, most notably the 2025 joint advisory from the American College of Lifestyle Medicine, the American Society for Nutrition, the Obesity Medicine Association, and The Obesity Society, highlight that nutrition practitioners are central to safe and sustainable GLP-1 use. Structured interventions emphasizing adequate protein intake, strategic fiber consumption, hydration strategies, anti-inflammatory diet patterns, supplementation, and resistance training can attenuate lean tissue loss, address GI side effects, and prevent deficiencies. Ongoing assessments, patient education regarding expected adverse effects, management strategies, and realistic goal setting are critical for adherence and continuation of therapy.

Challenges and Limitations in the Evidence Base

Despite the wealth of pharmacologic data on weight reduction outcomes, nutrition-specific outcomes remain poorly elucidated within the current body of scientific knowledge. Most studies are limited by a lack of quality information regarding dietary intake or baseline nutrient status biomarker data to evaluate longitudinal changes in patients. In addition, several nutrition‑relevant groups are rarely studied, such as older adults at risk of sarcopenia, individuals with eating disorders, people with malabsorption conditions, bariatric surgery patients, and adolescents. The impact of GLP-1s on many areas of high relevance to dietetic practice requires more robust and mechanistic investigation. The knowledge gaps constrain the ability to establish standardized nutrition care protocols or to generalize any findings across populations.

Future Direction

Future research should prioritize large-scale, longitudinal studies that integrate comprehensive dietary assessments, biomarker analysis, advanced body composition metrics (e.g., DXA, MRI), scintigraphy, and well-defined control groups to elucidate nutrient status and changes over the course of GLP-1 therapy for patients with obesity. Additionally, comparative studies between the different GLP-1 and GIP/GLP-1 drugs are needed to determine whether specific formulations exert distinct nutritional effects on their patients. Over time, the impact of routine dietitian involvement on clinical outcomes, adherence, and quality of life for GLP-1 patients could solidify their role as a standard, mandated component of GLP-1 therapy. Nutrition practitioners are uniquely positioned to provide tailored guidance that supports nutritional adequacy, metabolic adaptation, preservation of lean mass, and optimal health outcomes. Studies to evaluate the cost-effectiveness of integrating RDNs into GLP-1 care pathways will be essential in the development of standardized, scalable nutrition counseling programs to support GLP-1 RA patients.

As GLP-1 use expands globally, integrating structured nutrition care protocols will be essential not only for optimizing efficacy but also for safeguarding patient safety and long-term health. The evolving therapeutic landscape for individuals with obesity demands an evidence-based, multidisciplinary framework in which dietitians serve as critical partners in translating pharmacologic success into holistic, sustainable obesity care.

## Conclusions

GLP-1 receptor agonists have significantly advanced the treatment of obesity, achieving levels of weight loss previously unattainable with lifestyle intervention alone. However, their rapid clinical adoption has outpaced the development of nutrition-specific guidance, raising important concerns related to nutrient adequacy, lean mass preservation, and gastrointestinal tolerance. Evidence suggests that GLP-1-induced reductions in energy intake and shifts in food preferences may increase the risk of suboptimal nutrient intake, underscoring the critical role of dietitians in providing individualized, evidence-based medical nutrition therapy (MNT). Integrating RDN-led care into GLP-1 treatment models is essential to optimize outcomes, support adherence, and mitigate potential risks.

Despite promising outcomes, important gaps remain in the literature. Collectively, the clinical implications discussed throughout this paper highlight the need for structured interdisciplinary care models, and the proposed framework aims to provide healthcare professionals with an evidence-informed foundation for optimizing nutritional support and long-term outcomes in patients using GLP-1 medications. Future research should prioritize the development of standardized nutrition protocols, evaluate the long-term metabolic effects of pharmacologically induced energy restriction, and examine interactions with dietary patterns, body composition, bone health, and psychosocial factors. Additionally, considerations related to adaptive thermogenesis, weight maintenance post-cessation, and equitable access to both pharmacologic and nutritional care warrant further investigation. Strengthening reimbursement models to support dietitian integration will be critical to ensuring that the benefits of GLP-1 therapies translate into safe, sustainable, and equitable long-term health outcomes.
